# Costimulatory and coinhibitory immune checkpoint receptors in head and neck cancer: unleashing immune responses through therapeutic combinations

**DOI:** 10.1186/s41199-016-0013-x

**Published:** 2016-10-01

**Authors:** Ruth J. Davis, Robert L. Ferris, Nicole C. Schmitt

**Affiliations:** 1 Tumor Biology Section, Head and Neck Surgery Branch, National Institute on Deafness and Other Communication Disorders, National Institutes of Health, 10 Center Drive, Room 5B-39, Bethesda, MD 20892 USA; 2 Department of Otolaryngology, Hillman Cancer Center Research Pavilion, University of Pittsburgh, 5117 Centre Avenue, Room 2.26b, Pittsburgh, PA 15213-1863 USA; 3 Department of Immunology, Hillman Cancer Center Research Pavilion, University of Pittsburgh, 5117 Centre Avenue, Room 2.26b, Pittsburgh, PA 15213-1863 USA; 4 Cancer Immunology Programm, Hillman Cancer Center Research Pavilion, University of Pittsburgh Cancer Institute, 5117 Centre Avenue, Room 2.26b, Pittsburgh, PA 15213-1863 USA; 5 Department of Otolaryngology-Head and Neck Surgery, Johns Hopkins School of Medicine, 6420 Rockledge Drive, Suite 4920, Bethesda, MD 20817 USA

**Keywords:** Head and neck cancer, Immune checkpoints, Costimulatory receptors, CD137, CD40, OX40, PD-1, CTLA-4, Cetuximab, Immunotherapy

## Abstract

Head and neck squamous cell carcinoma (HNSCC) represents a model of escape from anti-tumor immunity. The high frequency of p53 tumor suppressor loss in HNSCC leads to genomic instability and immune stimulation through the generation of neoantigens. However, the aggressive nature of HNSCC tumors and significant rates of resistance to conventional therapies highlights the ability of HNSCC to evade this immune response. Advances in understanding the role of co-stimulatory and immune checkpoint receptors in HNSCC-mediated immunosuppression lay the foundation for development of novel therapeutic approaches. This article provides an overview of these co-stimulatory and immune checkpoint pathways, as well as a review of preclinical and clinical evidence supporting the modulation of these pathways in HNSCC. Finally, the synergistic potential of combining these approaches is discussed, along with an update of current clinical trials evaluating combinations of immune-based therapies in HNSCC patients.

## Background

Head and neck squamous cell carcinoma (HNSCC) is the sixth most common cancer in the world, affecting over 500,000 people each year [1]. While HPV-associated HNSCC responds well to standard anti-cancer therapies, five-year survival rates of carcinogen-induced HNSCC are 60 % or less [1]. This poor prognosis despite advances in chemotherapy, radiation, and surgical protocols highlights the need for treatments with greater efficacy in the HPV- population, and improved toxicity profiles for HPV+ patients. Advances in understanding the role of the immune system in preventing development and growth of HNSCC has led to renewed focus on immune-targeting therapies as a means of achieving these goals.

In a process termed immune surveillance, recognition of non-self antigens on tumor cells allows for their destruction by the host immune system [2]. The high frequency of p53 tumor suppressor loss in HNSCC leads to significant genomic instability and the generation of neoantigens, which can activate the immune system and attract infiltrates of effector T-lymphocytes and natural killer (NK) cells into the tumor [3–5]. These adaptive anti-tumor immune responses have been correlated with improved outcomes in many cancers, including HNSCC [6, 7]. However, in order for a clinically significant cancer to develop, the tumor must escape from this anti-cancer immunity through a variety of mechanisms [8]. HNSCC represents an ideal model for understanding and targeting these mechanisms of immune escape in order to unleash the full power of the immune response that can be induced by its characteristically high genetic alteration rate. On the other hand, HPV+ HNSCC is an excellent model of viral-induced cancer, in which oncoproteins such as E6 and E7 are by definition antigenic and therefore tumor development is predicated upon evasion of antiviral immunity [9].

**Fig. 1:**
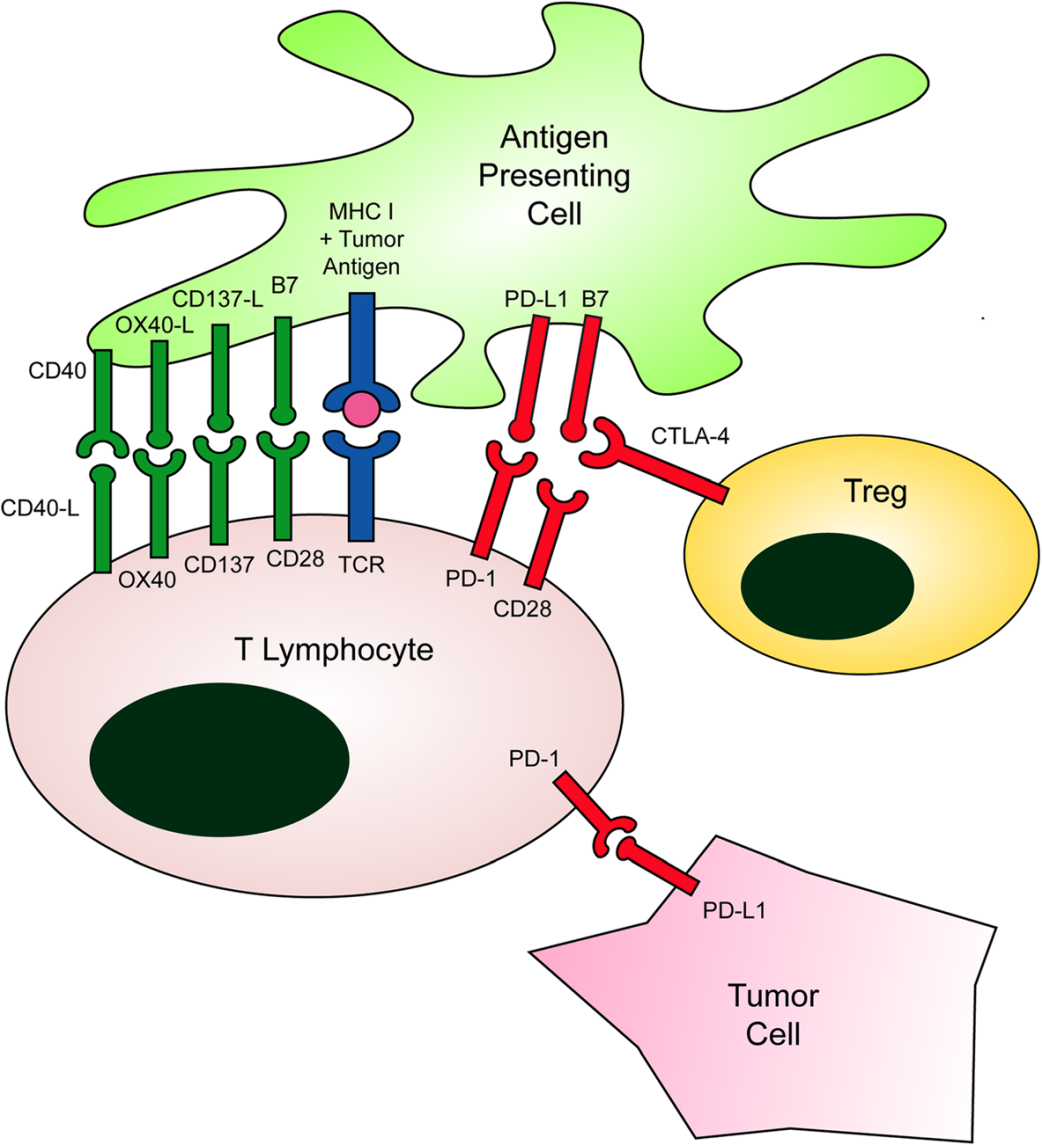
The Immune Synapse. The balance between costimulatory (*green*) and coinhibitory signals (*red*) alters the net stimulating effect of TCR signaling mediated through antigen presentation on MHC (*blue*). Adapted from Ferris RL, J Clin Oncol. 2015;33(29):3293–304

Once recruited to the tumor microenvironment, T-cells interact with antigen-presenting cells (APCs) at the “immune synapse,” and require two simultaneous signals from APCs before they can be activated to mediate their anti-tumor effects (Fig. 1). The first, “signal one,” occurs through interaction of the T-cell receptor (TCR) on the surface of the T-cell and a major histocompatibility complex (MHC) molecule presenting tumor antigen on the surface of an APC (Fig. 1, blue). The second, “signal two” is made up of interactions between co-stimulatory molecules on the surface of APCs and T-cells, such as B7 on the APC surface and CD28 on the T-cell (Fig. 1, green) [2]. Both of these signals must also occur in the context of a third signal made up of immune-activating cytokines such as IL-12, type I (IFNα/β) or type II (IFNγ) interferon [10, 11].

In contrast to these co-stimulatory molecules, the inhibitory “immune checkpoints” prevent T-cell activation. These checkpoints normally function to prevent exaggerated immune responses and subsequent autoimmune disease. However, HNSCC subverts this physiologic function in order to suppress tumor-directed immune activation. The two best known checkpoints include programmed death-1 (PD-1) and cytotoxic T-lymphocyte-associated protein 4 (CTLA-4), both of which are the targets of FDA-approved inhibitory antibodies [12].

Like other cancers, HNSCC tumor cells mediate immunosuppression in the tumor microenvironment (TME) through mechanisms including upregulation of PD-L1 expression and release of immunosuppressive factors [13]. In addition, recruitment or differentiation of immunosuppressive regulatory T cells (Tregs) and myeloid-derived suppressor cells (MDSCs) represent key mechanisms of immune escape. This review will focus on co-stimulatory receptors, inhibitory checkpoint receptors, and combination immunotherapies in HNSCC.

## Review

### Activating co-stimulatory receptors to enhance anti-tumor immune responses

As mentioned previously, the co-stimulatory receptors that make up “signal two” play a central role in the activation of tumor-fighting T-cells. Absence of this co-stimulatory signal can lead to induction of T-cell anergy or apoptosis and decreased strength of the immune response. In addition to the classically described CD28/B7, other key co-stimulatory interactions between T-cells and APCs, respectively, include CD137/CD137-L, OX40/OX40-L, and CD40-L/CD40 (Fig. 1). These costimulatory receptors are members of the tumor necrosis factor receptor superfamily. Decreased expression of CD137, OX40, CD27, and CD28 have been observed on T-cells derived from HNSCC patients compared to those from healthy controls, emphasizing the potential benefits of targeting these co-stimulatory pathways in this population [14–16]. Agonists of co-stimulatory receptors are currently under investigation in multiple trials for HNSCC and other malignancies. Liver toxicity and cytokine storm symptoms have been reported with costimulatory agonists, but these immune-related adverse events (irAEs) may be dose-dependent. Though extensive information on irAEs of these agents is not yet available, they appear to be well tolerated [17].

#### CD137

CD137 (also known as 4-1BB) is a costimulatory receptor expressed on the surface of activated T-cells, NK cells, and dendritic cells. When bound by its ligand (CD137-L) on the surface of APCs such as macrophages, dendritic cells, and B cells, trimerization of activated CD137 enhances proliferation, cytotoxic capacity, and survival of T-cells [18]. Stimulation of CD137 with an anti-CD137 monoclonal antibody (mAb) has been shown to induce T-cell mediated eradication of established solid tumors in mice [19]. Although anti-CD137 mAb has not been effective as a monotherapy in HNSCC models, it has been shown to synergize with chemoradiation in a model of HPV+ HNSCC to inhibit tumor growth [20, 21].

Two humanized monoclonal antibodies (mAb) against CD137 have been developed including urelumab (IgG4) and PF-05082566 (IgG2) [12]. These antibodies have been evaluated in early phase trials in melanoma, non-small cell lung cancer (NSCLC) and lymphoma, as monotherapy or in combination with rituximab [12]. Studies of CD137 mAb in combination with other immunotherapies are underway in solid cancers including HNSCC, and are discussed further below.

#### CD40-L

CD40-L expressed on the surface of activated CD4 T-cells binds to CD40 on APCs, playing a key role in the “helper” T-cell function to activate APCs to prime CD8 T-cells [22]. Expression of both CD40 and CD40-L decrease with increasing HNSCC stage, and surgical resection results in increased APC expression of CD40 [23]. These data implicate downregulation of this co-stimulatory pathway in HNSCC immune escape, with subsequent reversal following surgical resection of the tumor bulk. In addition to its expression on immune cells, CD40 has also been identified on HNSCC cell lines and human HNSCC tumors [24, 25]. The precise role of CD40 in this context is controversial, as ligation of CD40 has been shown to inhibit growth of HNSCC cell lines while also inhibiting cancer cell apoptosis and increasing secretion of proangiogenic cytokines [24, 25]. In vitro studies of agonistic CD40 mAb induced APC activation and maturation, and recombinant CD40L increased the ability of APCs to cross-prime naïve T-cells to tumor antigens [26, 27]. These data suggest a role for this approach in augmenting responses to tumor vaccines, which has been demonstrated in a murine solid tumor model [28].

A variety of CD40-targeting therapies have been developed, including agonistic mAbs and recombinant ligands. Although this approach has not been studied specifically in the HNSCC population, one HNSCC patient treated in a phase I trial of recombinant CD40-L experienced a durable and complete response [29]. Phase I trials of agonistic CD40 mAb alone and in combination with standard therapies have shown promise in a variety of solid tumors, encouraging further studies of this approach in combination with other immune-targeting therapies [30–32].

#### OX40

Like CD137, OX40 is a co-stimulatory molecule expressed on the T-cell surface that promotes T-cell proliferation, cytokine secretion and memory function when bound by its ligand (OX40-L) [12]. The relevance of OX40 to the local immune response in HNSCC has been demonstrated by the observation that close to 30% of T-cells within the tumor and tumor-draining lymph nodes of HNSCC patients expressed OX40, compared to none of the peripheral blood mononuclear cells (PBMCs) [33]. OX40 is also expressed on regulatory T-cells (Tregs), and appears to inhibit Treg-mediated immunosuppression [34]. Although not explicitly evaluated in HNSCC models, OX40 agonism has improved tumor-free survival in a number of solid tumor models through expansion of tumor-specific CD4+ T-cells [34]. An agonistic OX40 mAb was also shown to synergize with cytolytic therapy through apoptosis of Tregs and enhanced CD8 T-cell response [35]. In a sarcoma model, surgical resection followed by adjuvant anti-OX40 treatment resulted in improved survival and increased antigen-specific T-cell proliferation compared to surgical treatment alone [36].

A phase I trial of the murine agonistic anti-human OX40 mAb 9B12 in patients with refractory solid tumors demonstrated a mild toxicity profile and promising immunologic correlates (NCT01644968) [37]. Although no objective responses were observed, 12 out of the 30 patients experienced regression of at least one metastatic tumor deposit [37]. Additional therapies targeting OX40, including the anti-OX40 mAbs MEDI6469, MEDI0562, PF-04518600, and the OX40L fusion protein MEDI6383, are currently in phase I trials in patients with advanced solid tumors (NCT02205333, NCT02318394, NCT02315066, NCT02221960). In addition, based on the promising preclinical data combining anti-OX40 treatment with surgery [36], a phase Ib trial of OX40 agonistic mAb MEDI6469 prior to definitive surgical resection is currently recruiting patients with locoregionally advanced HNSCC (NCT02274155).

### Inhibiting immune checkpoint receptors to enhance anti-tumor immune responses

A reciprocal approach to agonism of co-stimulatory receptors is the inhibition of the immunosuppressive checkpoint receptors. These molecules are upregulated by immune activation, and serve a physiologic role by preventing excessive inflammation and autoimmune disease. However, when overexpressed in the TME, these checkpoints contribute to tumor-promoting immunosuppression, and therefore represent a promising target for disinhibiting immune responses against tumor cells and improving HNSCC patient outcomes. Immune-related adverse events (irAEs) may occur in patients treated with these drugs, including rash, gastrointestinal symptoms, thyroid disorders or autoimmune pneumonitis; however these irAEs are now easily recognized and treated with steroids and/or cessation of the drug in most cases [38].

#### PD-1/PD-L1

PD-1 is expressed by activated CD8 T-cells, NK cells, B cells, monocytes, and dendritic cells, and normally serves to prevent overactivation of the immune response [12]. However, chronic antigen exposure can lead to chronic upregulation of PD-1 and subsequent T-cell fatigue [39]. Ligation by PD-L1 inhibits activation signaling through the TCR. PD-L1 is expressed by the majority of HNSCC tumors, and blockade of PD-L1 has been shown to synergize with T-cell immunotherapy in an animal model of HNSCC [40]. CD8 T-cells derived from HPV+ HNSCC samples expressed high levels of PD-1, and HPV+ HNSCC cells were observed to express greater levels of PD-L1 compared to HPV- samples [41, 42]. In addition, infiltration of T-cells expressing PD-1 has been associated with a better prognosis in HPV+ disease, emphasizing the role of prior immune activation in patient prognosis [42].

A variety of antibodies have been developed against both PD-1 and PD-L1, including pembrolizumab (anti-PD1 mAb; FDA approved for HNSCC, melanoma and NSCLC) and nivolumab (anti-PD1; FDA approved for melanoma, NSCLC, and renal cell carcinoma). Numerous clinical trials targeting the PD-1/PD-L1 pathway have been extensively discussed in recent reviews [2, 13, 43]. Therefore we will only briefly highlight a few current late-phase trials in HNSCC, then further discuss checkpoint inhibitor combination trials below. Of note, the Keynote 40 and Keynote 48 phase III trials comparing pembrolizumab to standard of care are currently recruiting patients with recurrent or metastatic HNSCC who have failed platinum-based treatment (NCT02252042), or as first-line therapy (NCT02358031). The Keynote 55 phase II trial is evaluating pembrolizumab in HNSCC patients who have failed both platinum and cetuximab therapy (NCT02255097). Pembrolizumab was recently FDA approved based on long-term data from the KEYNOTE-012 trial including HNSCC patients with recurrent or metastatic HNSCC on or following platinum-based chemotherapy. KEYNOTE-012 showed an overall response rate of 17.7 %, median overall survival of 8.5 months, and 6-month progression-free survival (PFS) rate of 25 %; 12 % of patients had grade 3–4 adverse events [44]. The Checkmate 141 phase III trial comparing nivolumab to investigator’s choice in patients with recurrent or metastatic, platinum-refractory HNSCC (NCT02105636) was stopped early due to significant survival benefit including a 30 % reduction in risk of death and doubling of one-year survival from 17 to 36 % [45]. Median overall survival in Checkmate 141 was 7.5 months with nivolumab vs. 5.1 months for investigator’s choice therapy; overall response rate ranged from 18–33 %, with higher response rates noted in patients whose tumors expressed higher levels of PD-L1 [46]. Similar to KEYNOTE-012, in Checkmate 141 the rate of grade 3–4 adverse events was 13 % [45, 46].

#### CTLA-4

CTLA-4 is transiently expressed by activated T-cells upon binding of an antigen-bearing MHC molecule to the TCR, thereby limiting exaggerated immune responses [47]. CTLA-4 is also constitutively expressed on the surface of Tregs in the HNSCC microenvironment [48]. In animal models, CTLA-4 expression was necessary to the immunosuppressive function of Tregs, and conditional knockout of CTLA-4 in Tregs protected from tumor development [49]. CTLA-4 binds B7 ligands CD80 and CD86 with higher affinity than CD28, thereby competitively inhibiting “signal two” in the T-cell activation cascade [47]. Preclinical studies in solid tumor models demonstrated regression of established tumors and the rejection of further tumor challenge following anti-CTLA-4 mAb treatment [50].

Since that time, two humanized anti-CTLA-4 mAbs, ipilimumab (IgG1) and tremelimumab (IgG2), have been developed and evaluated in phase III trials in advanced melanoma [47]. Based on results of two phase III trials of ipilimumab demonstrating enhanced survival and improved tumor responses, ipilimumab became the first FDA-approved checkpoint inhibitor in 2011 [51, 52]. For patients with platinum-refractory, recurrent or metastatic, PD-L1-negative HNSCC, an ongoing phase II/III study includes tremelimumab and durvalumab (anti-PD-L1) as separate monotherapies or in combination (NCT02319044). Trials combining multiple checkpoint inhibitors are further discussed below.

#### LAG-3

LAG-3 is another inhibitory checkpoint that is expressed on the surface of Tregs in HNSCC patients [53]. LAG-3 has been identified as a key regulatory molecule involved in prevention of autoimmune disease, as well as the development of tumor tolerance [54, 55]. Knockout of *LAG-3* in murine models has been shown to reduce the immunosuppressive activity of Tregs, and conversely ectopic expression of LAG-3 has been shown to confer immunosuppressive capacity upon CD4 T-cells [56]. In addition to playing a role in the immunosuppressive functions of Tregs, LAG-3 expression has also been observed on effector CD8 T-cells at the immunologic synapse [57]. Anti-LAG-3 mAb treatment in solid tumor models has shown success in inhibiting primary tumor growth through activation of antigen-specific T-cells in the TME [55]. In murine solid tumor models, LAG-3 and PD-1 co-expression has been identified on the surface of TILs, and combination anti-LAG-3 and anti-PD-1 antibody treatment cured the majority of established tumors in mice [58]. Early phase clinical trials evaluating anti-LAG-3 mAb in combination with other checkpoint inhibitors are reviewed in Table 1.

**Table 1:** Current Combination Immunotherapy Trials Including HNSCC Patients

Targets	Treatments	Phase	Clinical Trial ID	Patient Eligibility	Status
Costimulatory/Checkpoint Combinations					
CD137 (4-1BB) PD-L1	PF-05082566 + Avelumab	Ib/II	NCT02554812	Advanced/metastatic solid tumors	Recruiting
CD137 (4-1BB) PD-1	PF-05082566 + Pembrolizumab	I	NCT02179918	Advanced/metastatic solid tumors	Recruiting
OX40 PD-L1	MEDI6383 +/−Durvalumab	I	NCT02221960	Recurrent or metastatic solid tumors	Recruiting
OX40 CTLA-4 PD-L1	MEDI6469 Alone, + Tremelimumab, or + Durvalumab	Ib/II	NCT02205333	Advanced solid tumors	Ongoing, not recruiting
CD27 PD-L1	Varlilumab + Atezolizumab	I/II	NCT02543645	Advanced cancers including HNSCC	Recruiting
CD27 PD-1	Varlilumab + Nivolumab	I/II	NCT02335918	Advanced solid tumors	Recruiting
Checkpoint/Checkpoint Combinations					
CTLA-4 B7-H3	Ipilimumab + MGA271	I	NCT02381314	Advanced/metastatic B7-H3+ HNSCC, melanoma, or NSCLC	Recruiting
CTLA-4 PD-L1	Tremelimumab + Durvalumab	III	NCT02551159	HNSCC with no prior chemotherapy	Recruiting
CTLA-4 PD-L1	Tremelimumab + Durvalumab	I	NCT02262741	Recurrent or metastatic HNSCC	Recruiting
CTLA-4 PD-L1	Tremelimumab + Durvalumab (monotherapy or combination)	II	NCT02319044	Recurrent or metastatic HNSCC	Ongoing, not recruiting
CTLA-4 PD-L1 Vaccine	Tremelimumab + Durvalumab + PolyICLC	I/II	NCT02643303	Advanced solid tumors including HPV- HNSCC or HPV+ HNSCC after prior treatment failure	Not yet recruiting
PD-L1 CTLA-4	Durvalumab +/−Tremelimumab	III	NCT02369874	Recurrent or metastatic HNSCC	Recruiting
PD-1 B7-H3	Pembrolizumab + MGA271	I	NCT02475213	B7-H3+ advanced HNSCC	Recruiting
PD-L1 HPV E7	Durvalumab + ADXS 11–001	I/II	NCT02291055	Recurrent or metastatic HPV-associated HNSCC	Ongoing, not recruiting
LAG-3 PD-1	BMS-986016 +/− Nivolumab	I	NCT01968109	Advanced solid tumors	Recruiting
LAG-3 PD-1	LAG525 +/− PDR001	I/II	NCT02460224	Advanced solid tumors	Recruiting
TIM-3 PD-1	MBG453 +/−PDR001	I/II	NCT02608268	Advanced solid malignancies	Recruiting
Cetuximab Combinations					
CD137 (4-1BB)	Urelumab + Cetuximab	Ib	NCT02110082	Advanced/metastatic HNSCC or CRC	Ongoing, not recruiting
CTLA-4	Iplimumab + Cetuximab + IMRT	Ib	NCT01860430, NCT01935921	Stage III-IVB HNSCC p16- or intermediate-risk p16+	Recruiting
TLR8	Cetuximab + SOC Chemo (CDDP + 5-FU) +/− VTX-2337	II	NCT01836029	Recurrent or metastatic HNSCC	Ongoing, not recruiting
TLR8	Cetuximab + VTX-2337 window of opportunity before surgery	Ib	NCT02124850	Stage II-IVA resectable HNSCC	Recruiting

#### TIM-3

TIM-3 represents an additional inhibitory checkpoint that has been implicated both in the immunosuppressive function of Tregs and in the exhaustion of effector T cells in the TME. Elevated expression of TIM-3 has been observed on intratumoral Tregs derived from patients with HNSCC and non-small cell lung cancer, and has been observed to correlate with worse clinical outcomes [53, 59]. TIM-3 has also been implicated in the exhaustion of effector T cells through upregulation of TCR signaling [60]. Anti-TIM-3 mAbs have been shown to modestly inhibit solid tumor growth in murine models, and have induced more impressive control of tumor growth in combination with CTLA-4 and PD-1 targeting therapies [61, 62].

#### B7-H3

Initially identified as a co-stimulatory receptor of T cell function [63], B7-H3 has since been described as a co-inhibitory checkpoint expressed in a variety of tumor types [64, 65]. Although the specific immunological role of B7-H3 in cancer remains controversial, B7-H3 expression has been correlated with poor prognosis in multiple cancer types, including HNSCC [66]. Antibodies targeting the B7-H3 molecule have been shown to exhibit antitumor activity in solid tumor models with surface expression of B7-H3 [67]. Early phase trials combining these agents with other checkpoint inhibitors are currently underway (Table 1).

### Combination immunotherapies for maximal enhancement of anti-tumor immune responses

Although many of the above mentioned immunotherapy approaches have shown significant efficacy in certain patients, there is room for improvement with regards to expanding response rates. Current efforts focus on the rational combination of immunotherapy approaches in order to increase the breadth and depth of patient responses (Table 1). It is important to note, however, that combination immunotherapies may increase the frequency and/or severity of immune-related adverse events [38].

#### Cetuximab

**Fig. 2:**
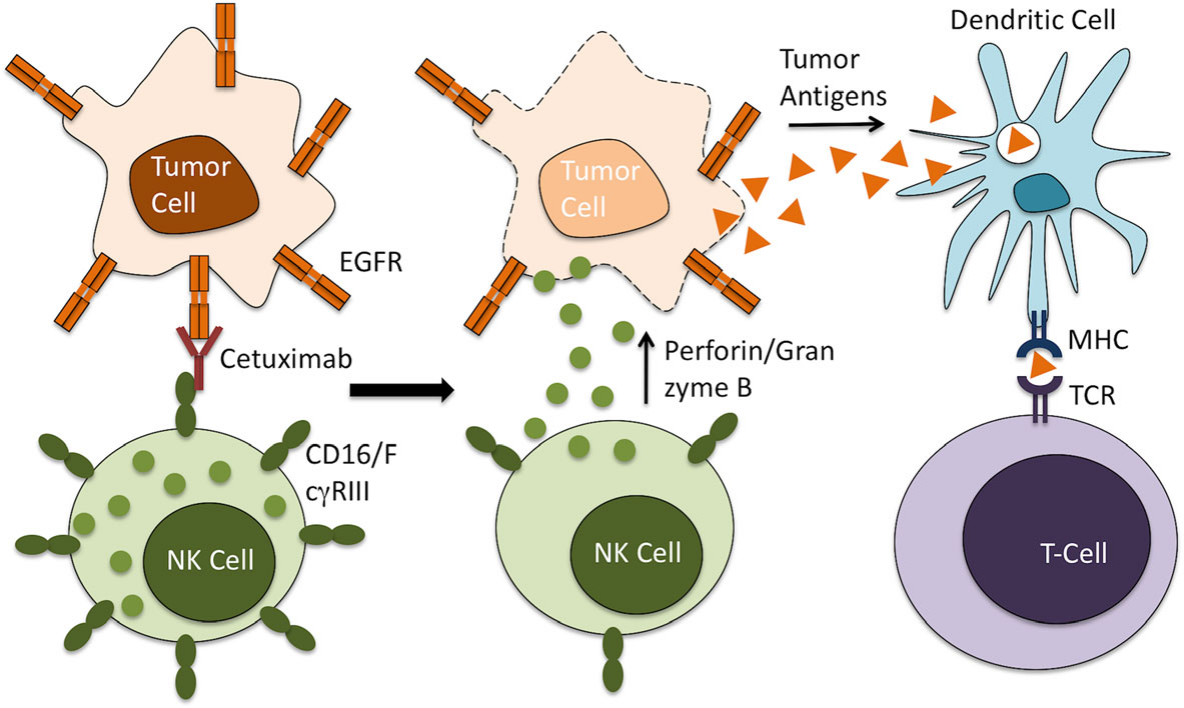
Cetuximab-Mediated ADCC. The Fab portion of Cetuximab binds to EGFR on the surface of tumor cells, while its Fc region binds to the Fc receptor CD16/FcγRIII on the NK cell surface. This leads to NK cell activation and release of cytolytic granules containing perforin and granzyme B that result in tumor cell lysis and release of tumor antigen. This tumor antigen is subsequently presented on APCs to activate antigen-specific T-cells

Cetuximab is a human-mouse chimeric IgG1 antibody against epidermal growth factor receptor (EGFR) that is FDA approved as a monotherapy for recurrent/metastatic HNSCC, in combination with radiation therapy for advanced HNSCC, and in combination with chemoradiation for recurrent/metastatic HNSCC [13]. Although more than 80 % of HNSCC tumors overexpress EGFR, only 10-20 % of patients respond to cetuximab treatment [68]. In patients who respond, cetuximab is thought to mediate part of its effect through inhibition of EGFR signaling and downstream proliferation signals. However, evidence suggests that much of the therapeutic effect of cetuximab is derived from activation of NK cells and antibody-dependent cell-mediated cytotoxicity (ADCC) [43]. Extracellular binding of cetuximab to EGFR exposes the constant region (Fc) of cetuximab to binding by the activating Fc receptor (CD16/FcγRIII) expressed on NK cells. This activation signal induces ADCC mediated by NK cells resulting in tumor cell lysis (Fig. 2). The variability of patient response to cetuximab is thought to be in part due to polymorphisms in NK cell FcγRIII, which lead to variation in its affinity for the Fc region of cetuximab [69].

In addition to mediating ADCC, cetuximab-activated NK cells have been shown to promote maturation of APCs and the development of an adaptive immune response [68]. Recent evidence also suggests that cetuximab treatment decreases the function of immunosuppressive myeloid cells [70]. Increased numbers of monocytic MDSCs were observed in patients who did not respond to cetuximab therapy, suggesting potential for improving responses through combining cetuximab with MDSC-targeting treatments [70]. Given the low response rate to cetuximab as a monotherapy or in combination with standard therapies, recent efforts have focused on combining cetuximab with additional immunotherapies to enhance ADCC.

Human NK cells have been shown to upregulate surface expression of CD137 following exposure to cetuximab and EGFR-expressing cell lines [71]. In preclinical studies, sequential treatment with cetuximab followed by anti-CD137 mAb eradicated established tumors in an NK-cell dependent manner [71]. In addition to this enhancement of ADCC, preclinical evidence also supports a mechanistic role for the adaptive immune “vaccinal effect.” Mice previously cured with this combination therapy rejected rechallenge with both EGFR-positive and negative cell lines, which supports immunologic memory and epitope spreading [72]. Based on this promising preclinical data, a phase Ib trial combining cetuximab with the anti-CD137 mAb urelumab is currently underway (NCT02110082).

In addition to FcγRIII polymorphisms, proposed mechanisms of resistance to cetuximab-mediated ADCC include an increase in the number of Tregs within the HNSCC TME following cetuximab treatment [73]. These CTLA-4+ Tregs were shown to suppress cetuximab-mediated ADCC, and their increased numbers correlated with poor patient prognosis [73]. *Ex vivo* treatment of HNSCC tumor-infiltrating lymphocytes with the anti-CTLA-4 mAb ipilimumab depleted Tregs and restored NK cell-mediated ADCC [73]. Based on this promising preclinical data, two phase Ib studies combining ipilimumab with intensity modulated radiation therapy (IMRT) and cetuximab are currently recruiting patients with untreated advanced HNSCC (NCT01860430, NCT01935921).

#### Toll like receptor agonists

Toll-like receptors (TLRs) are transmembrane receptors that recognize microbial invasion and respond through activation of the innate immune system [74]. TLR7 and TLR8 have been particular targets for improving anti-cancer immunity. An early topical TLR7/8 agonist, imiquimod, is FDA approved for actinic keratosis and basal cell carcinoma. In addition, novel stabilized immune-modulatory RNA (SIMRA) compounds are also under study for their dual TLR7/8 agonism [75]. However, recent development of a potent and selective TLR8 agonist has focused attention on this endosomal TLR that is naturally activated by viral single-stranded RNA. Stimulation of TLR8 results in activation of dendritic cells and macrophages and subsequent secretion of immune-activating cytokines. TLR8 signaling has also been implicated in reversal of Treg function [76]. VTX-2337, a TLR8 agonist, has been shown to induce TNFα and IL-12 secretion by monocytes and myeloid dendritic cells, in addition to increasing NK cell cytotoxicity and secretion of IFNγ [77]. VTX-2337 has also been reported to enhance rituximab and trastuzumab-induced ADCC in lymphoma and breast cancer cells lines, respectively [77]. Subsequent preclinical studies using PBMCs from healthy individuals and HNSCC patients demonstrated the ability of VTX-2337 to enhance cetuximab-mediated ADCC against HNSCC cells [78].

Based on these promising preclinical data regarding selective TLR8 agonism, VTX-2337 was studied in a phase I trial in advanced solid tumors [79]. This trial demonstrated clinical tolerability in addition to increases in plasma levels of immune-activating cytokines G-CSF, monocyte chemoattractant protein-1, macrophage inflammatory protein-1β, and TNFα when administered at higher doses. Based on this information, phase II placebo-controlled trials of combination therapy with VTX-2337 have been initiated, including a comparison of chemotherapy + cetuximab + VTX-2337 to chemotherapy + cetuximab alone in recurrent or metastatic HNSCC (NCT01836029). In addition, a phase Ib study of neoadjuvant cetuximab + VTX-2337 vs. cetuximab + VTX-2337 + nivolumab is currently recruiting patients with stage II-IVA surgically resectable HNSCC (NCT02124850).

#### Checkpoint inhibitors

Although the degree and durability of response to checkpoint inhibitor monotherapy has been impressive, objective response rates remain low. For example, preliminary data from the KEYNOTE-012 expansion cohort showed an objective response in 18.2 % of recurrent/metastatic HNSCC patients treated with pembrolizumab monotherapy [80]. For this reason, much attention is currently directed towards combining checkpoint inhibitors with a variety of immune-based therapies to achieve higher response rates in both preclinical and clinical studies (Table 1).

Signaling through various immune checkpoints and downregulation of costimulatory receptors each represent a distinct mechanism of tumor-mediated immunosuppression. Combining inhibitors that target different checkpoints is a logical strategy to generate synergy and target potential mechanisms of resistance to therapy. For example, melanoma patients with high PD-L1 expression did not respond to anti-CTLA4 mAb and radiation, implicating PD-1/PD-L1 signaling in this resistance (NCT01497808) [81]. However, in preclinical studies of melanoma, combined targeting of CTLA-4 and PD-1 more than doubled the rate of tumor rejection and increased tumor-infiltrating T-cells while reducing Tregs and MDSCs in the TME [82]. Synergism has also been described between antibodies targeting PD-1 and TIM-3 [61], and PD-1 and LAG-3 [58] in solid tumor models, leading to current clinical trials evaluating these combinations in patients with advanced solid tumors (NCT02608268, NCT01968109, NCT02460224).

A phase I trial of combined nivolumab (anti-PD-1) and ipilimumab (anti-CTLA-4) in advanced melanoma showed an overall response of 40 % and objective responses in 53 % of patients treated with the maximal tolerated dose [83]. These outcomes exceed responses seen with either drug as a monotherapy. Preliminary results from another phase I trial combining durvalumab (anti-PD-L1) and tremelimumab (anti-CTLA-4) in NSCLC patients showed an overall response rate of 25 %, and the interesting finding that this efficacy did not depend on PD-L1 expression in the tumor [84]. Many further studies combining checkpoint inhibitors with one another and with co-stimulatory molecules are currently underway and summarized in Table 1.

In addition, simultaneous agonism of co-stimulatory pathways and antagonism of inhibitory checkpoints allows one to “step on the gas while taking the foot off the brakes.” In solid tumor models, combined treatment with agonistic anti-OX40 mAb and anti-CTLA-4 mAb improved survival and induced tumor regression through expansion of effector CD8 T-cells [85]. Combined targeting of OX40 and PD-L1 or OX40 and CTLA-4 is currently under study in early phase trials in advanced solid tumors (NCT02221960 & NCT02205333). Studies in solid tumor models also demonstrated synergy between mAbs targeting CD137, PD-1, and CTLA-4 [86]. Based on these data, two current phase I/II trials are evaluating the combination of anti-CD137 and anti-PD-1/PD-L1 mAb in advanced solid tumors (NCT02554812 & NCT02179918).

#### Combination of immunotherapies with standard or targeted therapies

In addition to combination immunotherapies for HNSCC, other promising strategies under investigation include the combination of these agents with standard-of-care or targeted therapies. Studies in HNSCC and other cancer types suggest that radiotherapy, cisplatin chemotherapy, and other cytotoxic drugs may enhance anti-tumor adaptive immunity in the TME [87–90]. As a result, radiation and cisplatin may also increase immune checkpoint expression [89–92]. These findings suggest a strong rationale for combining radiation and/or chemotherapy with checkpoint inhibitors and other immune therapies, and such combinations are currently under study in multiple clinical trials.

The Cancer Genome Atlas and other studies have revealed specific genomic alterations in HNSCC that may be targeted by specific therapies [93]. Although cetuximab is so far the only targeted agent that is FDA-approved for HNSCC, a myriad of targeted agents are under investigation in preclinical and clinical studies of HNSCC. Future treatment strategies are likely to utilize combinations of targeted agents with immune and standard therapies.

#### HPV-specific immunotherapies

As mentioned above, HPV-associated HNSCC represents distinct mechanisms of antiviral immune escape, and this disease entity also may benefit from specific antiviral immunotherapies. Since patients with HPV-associated disease generally have an excellent prognosis and high cure rates, newer therapeutic strategies have focused on improving upon long-term toxicities seen with current therapies. Therapeutic vaccines and adoptive transfer of immune cells have been studied in HPV-associated HNSCC [2] and will be combined with surgery, chemoradiation, targeted therapies or other immune therapies in ongoing and future trials. Trials of combination immunotherapies specific to patients with HPV-associated disease are detailed in Table 1.

## Conclusions

Recent advances in understanding the balance between costimulatory and inhibitory immune pathways at the immune synapse have encouraged interest in re-directing these signals from tumor-promoting immunosuppression towards tumor-fighting immunity. With the development of an array of costimulatory agonists and checkpoint inhibitors, these immune-based strategies have become a focal point for research in many cancers, including HNSCC. In addition to focusing on clinical application of these novel immunotherapies, much work is underway to investigate mechanisms of resistance in those patients who do not achieve durable responses. Rational design of combination strategies represents a promising approach to target resistance, while care must be taken to avoid immune over-activation and serious autoimmune consequences.

## Abbreviations

APC: Antigen-presenting cell
CTLA-4: Cytotoxic T lymphocyte-associated protein 4
EGFR: Epidermal growth factor receptor
HNSCC: Head and neck squamous cell carcinoma
HPV: Human papillomavirus
IMRT: Intensity modulated radiation therapy
LAG-3: Lymphocyte-activation gene 3
mAb: Monoclonal antibody
MDSC: Myeloid-derived suppressor cell
MHC: Major histocompatibility complex
NSCLC: Non small-cell lung cancer
PBMC: Peripheral blood mononuclear cell
PD-1: Programmed death-1
PD-L1: programmed death-1 ligand
TCR: T-cell receptor
TIM-3: T-cell immunoglobulin mucin 3
TLR: Toll-like receptor
TLR: Toll-like receptor
TME: Tumor microenvironment
Treg: Regulatory T-cell
